# Complex Expressions of Power. Aspects of Gender in/Equality in Violent Intimate Relationships in Sweden

**DOI:** 10.1177/10778012251348433

**Published:** 2025-06-12

**Authors:** Maria Wemrell, Linda Hiltunen

**Affiliations:** 1 Department of Social Work, 8180 Linnaeus University, Växjö, Sweden; 2 Unit for Social Epidemiology, Department of Clinical Sciences, 5193 Lund University, Malmö, Sweden; 3 Department of Social Studies, 8180 Linnaeus University, Växjö, Sweden

**Keywords:** intimate partner violence against women, gender equality, power relations, Nordic paradox, Sweden

## Abstract

Despite high country-level gender equality, intimate partner violence against women (IPVAW) is common in Sweden. In discussion about this apparent paradox, the complexity of gender in/equality has been highlighted. Drawing on the domains considered in the EIGE gender equality index (work, money, knowledge, time, power, and health), and based on thematic analysis of semi-structured interviews with 23 women exposed to IPVAW, this study investigates experiences of different aspects of gender in/equality in violent partnerships. The violence was described as having influenced and involved all these domains of life. We conclude that gendered power dynamics remain central to IPVAW in Sweden.

## Introduction

Intimate partner violence against women (IPVAW) is a global public health and human rights issue, which is typically perceived to be associated with gender inequality (e.g., Council of Europe, 2011). Still, while Sweden has been rated as one of the EU's most gender-equal countries (e.g., EIGE, 2017a, 2024), survey data indicate that the IPVAW prevalence in Sweden is similar to or higher than in other EU states (FRA, 2014; FRA, EIGE & Eurostat, 2024). This apparently contradictory co-existence of high levels of country-level gender equality and of IPVAW found in Sweden and neighboring Nordic countries has been called the Nordic paradox (Gracia & Merlo, 2016). In discussions about this supposed paradox (Gracia & Merlo, 2016; Wemrell et al., 2020, 2022), the complex nature of gender in/equality and its links with IPVAW have been highlighted. The association between IPVAW and gender equality has also been placed in question, in favor of other causal or risk factors.

This study investigates women's experiences of gender equality and inequality in violent partnerships in Sweden, based on interviews with 23 survivors of IPVAW. Drawing on the six domains considered in the European Institute for Gender Equality's (EIGE) gender equality index (GEI) (EIGE, 2017a, 2024)—work, money, knowledge, time, power, and health—we analyze how these aspects of life were described in relation to IPVAW.

### IPVAW, Gender Equality, and the Nordic Paradox

IPVAW can be defined as physically, sexually, psychologically, or economically coercive acts against women by their current or former partners (UN, 2006), including violence occurring online or through information and communications technology (OHCHR, 2018), without their consent. While the causes of IPVAW are typically seen to be complex (cf. Heise, 1998), in research and policy IPVAW is commonly understood as being tied to gender inequality (e.g., Council of Europe, 2011; Dobash & Dobash, 1979). The EIGE (2017a) notes, for example, that IPVAW is both a driver and an effect of gender inequality, and a form of inequality in and of itself. Meanwhile, the EIGE GEI, which is a composite indicator assembling data pertaining to the domains of time, money, knowledge, time, power, and health, has rated Sweden as the EU's most gender-equal country since 2005 (e.g., EIGE, 2017a). In 2024, Sweden had the top score of 82.0, on the 1–100 scale, the EU-average being 71.0 (EIGE, 2024). The EIGE GEI has been critiqued for combining measures of gender gaps and of absolute achievements, to the disadvantage of low-income countries (Schmid & Elliot, 2023), but an adjusted rating based only on gender gaps also gave Sweden the top score (Permanyer, 2015). IPVAW is tied to gender inequality in Swedish political discourse (Prop, 2005/06:155; SOU, 2015:55) and the eradication of men's violence against women is a national gender equality goal (Skr, 2016/17:10). Internationally, Sweden has been categorized as progressive with regards to IPVAW law and policy (GREVIO, 2019). Nevertheless, an EU-wide survey (FRA, 2014) found that 28% of women in Sweden—above the EU average of 22%—reported exposure to physical or sexual IPVAW. In a more recent study (FRA, EIGE & Eurostat, 2024), 31% of women in Sweden reported having experienced physical or sexual IPVAW, and when psychological violence was added the number rose to 48.2%. This can be compared to the respective EU averages of 17.7% and 31.8%.

The association between high levels of IPVAW and of gender equality among countries in the EU (FRA, 2014) has weakened in re-analyses considering individual-level variables (Ivert et al., 2020) and attitudinal and other contextual factors (Humbert et al., 2021) or separating ongoing and past violence (Permanyer & Gomez-Casillas, 2020) or levels of coercive control (Nevala, 2017). The potential effects of reporting biases on IPVAW prevalence rates have also been discussed (Humbert et al., 2021; Wemrell et al., 2022), although psychometric analyses of the FRA data did not find such differences between countries (Gracia et al., 2019; Martín-Fernández et al., 2020). A remaining issue is that data from Nordic countries were gathered by telephone rather than in face-to-face interviews, as in other nations, in the FRA’s (2014) study. In sum, it may be that IPVAW is not actually more common in Sweden than in other EU states. A high IPVAW prevalence in Sweden remains, however, not least in young age groups, as also indicated by a range of other survey studies (e.g., Korkmaz et al., 2021; NCK, 2014; Strid et al., 2023).

Suggested explanations for the comparatively high IPVAW prevalence in Nordic nations (Gracia & Merlo, 2016; Wemrell et al., 2020) have include alcohol consumption patterns. While some studies affirm a connection between IPVAW perpetration and use of alcohol (e.g., Norén et al., 2025), others question the strength of the correlation (Gil-González et al., 2006; Grzyb, 2024; Wemrell et al., 2022) or point to the impact of alcohol in combination with other factors, such as expressions of dominance and control (Peralta et al., 2010). Another suggested explanation (Gracia & Merlo, 2016) is disproportionally high prevalence rates in some population groups, such as immigrated ones, due to discord between cultural norms in such groups and the majority population. While some studies find no systematic IPVAW prevalence differences between women born in Sweden or elsewhere (e.g., BRÅ, 2014; Lundgren et al., 2002; Nybergh et al., 2013), others indicate that groups of foreign-born women are indeed exposed to a high risk of IPVAW by men born in countries including Sweden (e.g., Fernbrant et al., 2016; Strid et al., 2021). It is very important that the needs of women in such groups are addressed, while not feeding into culturalizing or xenophobic understandings of violence as primarily and essentially existing among persons with immigration backgrounds rather than among Swedish-born people (e.g., GREVIO, 2019, 2024; Karlsson et al., 2021; Wemrell et al., 2019). However, the extent to which IPVAW in specific groups can explain higher prevalence rates in Nordic countries can be strongly questioned, for example, due to such rates having been high prior to more recent immigration waves (Lundgren et al., 2002; Wemrell et al., 2022). Further suggested explanations for high IPVAW prevalence in Nordic countries include pronounced individualism, lacking communication about emotion (Wemrell et al., 2022) and social isolation (Castro et al., 2024).

Apart from this, discussions about the Nordic paradox (Gracia & Merlo, 2016; Wemrell et al., 2022) have pointed to potential unintended consequences of relative gender equality, i.e., backlash effects, and to the complexity of gender equality.

#### The Complexity of Gender Equality and its Link with IPVAW

The suggestion that backlash effects may be relevant to understanding the Nordic paradox relates to the long-standing co-existence of two hypotheses on the nature of the link between gender equality and IPVAW and other forms of violence against women (VAW). Both have found support in empirical research. The amelioration hypothesis, which aligns with a liberal feminist outlook (Gómez-Casillas et al., 2023), posits that gender equality mitigates VAW. Thus, when gender equality increases, the latter is expected to decrease. Conversely, the backlash hypothesis, more aligned with radical feminism (Gómez-Casillas et al., 2023), links VAW to retaliation effects due to men's (perceived) loss of power due to advances towards gender equality (e.g., Franklin & Menaker, 2014).

Studies showing associations going in both directions (Kearns et al., 2020; Whaley & Messner, 2002) suggest that effects of gender equality on IPVAW are not straightforward. Some indicate that while VAW is likely to increase during early advances toward gender equality, once the latter has become more normative it shifts to being a protective factor (e.g., Vieraitis et al., 2015). However, in European countries with less traditional gender roles, rates of lethal VAW have been found to be higher (Stamatel, 2014). van Vugt and Pop (2022) also observed that women in the EU with higher income or education than their partners were more likely to report IPVAW. A higher risk of IPVAW has similarly been seen among women with higher income or education than their partners in Norway (Bjelland, 2014), and with high income in Sweden (Ericsson, 2020). A Swedish study of backlash narratives on the individual level found that IPVAW was described as being triggered by women's resources, agency, resistance, or breaking with traditional gender norms, or by the partner's perceived feeling of subordination (Wemrell, 2023).

Alongside the concept of backlash, resource theory (Goode, 1971), or relative resource theory (Macmillan & Gartner, 1999), posits that men with few socioeconomic resources, or fewer than their partners, may use violence as “the ultimate resource” (Allen & Straus, 1980) for control. That is, women with higher income, education, or employment status may be at increased risk, due to the man's dominant status being challenged, particularly if the man adheres to traditional gender norms (Atkinson et al., 2005).

Indicating a complex relationship between women's empowerment and their risk of IPVAW exposure (also e.g., Ince-Yenilmez, 2022; Vyas & Watts, 2009), Xie et al. (2012) found that while absolute increases in women's employment and income reduced their exposure to violence, their rising labor participation relative to men's was associated with a heightened risk. In a Swedish study of violence against older women (Ahnlund et al., 2020), lower education was associated with a lower prevalence, while experiencing economic problems was tied to a higher risk. Studying lethal VAW, in relation to the domains of the EIGE GEI, Stamatel (2018) found that while financial gender equality was associated with higher levels of femicide, health equality was tied to reduced risk and other domains showed no significant correlations. Gómez-Casillas et al. (2023) observed that gains in women's economic status decreased IPVAW, but backlash effects occurred when the overall status of women increased relative to that of men. They also noted that findings on backlash versus ameliorative effects were sensitive to which IPVAW or gender equality indicators that were used. In sum, increasing gender equality in one domain may not eliminate inequalities in others, as gender relations should be conceptualized as a complex phenomenon (e.g., Gartner et al., 1990).

Compared to other gender equality indexes, the EIGE GEI includes a wide range of dimensions of in/equality, including unpaid work (Schmid & Elliot, 2023). This is clearly relevant, as the division of domestic labor is a central aspect of societal power relations between men and women (e.g., Hartmann, 1981; Walby, 1990). Still, the degree to which the EIGE GEI reflect all aspects of gender in/equality relevant to IPVAW has been questioned. IPVAW professionals in Sweden (Wemrell et al., 2022) noted that while much progress has been made regarding women's income, education, and political representation, development in the private sphere, particularly in heterosexual relationships, is more questionable. Discussing gender norms and relational power dynamics, some referred to Swedish gender equality as partially being a surface-level phenomenon (Wemrell et al., 2022). Relatedly, the UN Special rapporteur on VAW (Ertürk, 2007) argued that while advances in gender equality had been made in public arenas in Sweden, these seemed to have halted at the doorsteps of private homes. This aligns with Nousiainne et al.'s (2013) observation that efforts toward gender equality in the public sphere do not necessarily address power relations in the private realm.

Research has indeed indicated a disjuncture between advancing gender equality in policy and the public sphere in Sweden and maintained gendered power imbalances in the private arena where IPVAW occurs (e.g., Gunnarsson, 2013; Nyman et al., 2018). Studies have pointed to how norms of masculinity, femininity, and heterosexual relationships can create relational dynamics sliding towards and into IPVAW (Wemrell et al., 2019), and to how tensions due to the co-existence of gender equality norms with more traditional ones (Wemrell et al., 2022), or due to expectations of or efforts towards gender equality (Wemrell, 2023), can trigger IPVAW. Such findings can be related to feminist analyses noting that patriarchy has both structural and ideological components, the former allowing men to maintain positions of power and the latter rationalizing it (DeKeseredy, 2021). Efforts towards equality which shift structural conditions, for example through increasing women's political representation, do not self-evidently address ideological, including psychological, elements conducive to VAW (Hunnicutt, 2009). This has been pointed out as a possible explanation for the co-existence of relative gender equality and violence against, or coercive control of, women (Pease, 2019; Stark, 2023).

Relatedly, in a discussion of low reported levels of VAW in relatively gender-unequal Poland (FRA, 2014), Grzyb (2024) points to sociocultural aspects of gender relations. Exploring factors such as resistance to gender equality policies pushed by the communist regime having formed part of a nationalist struggle which emphasized traditional female roles and myths of such, and strong female figures being strongly present in society all the while hegemonic masculinity was not seriously challenged, she suggests that patriarchy in Poland may place women in a position less fraught with risk of IPVAW than in Western EU states. Thus, she indicates that sociocultural factors and norms can be more relevant for understanding IPVAW prevalence rates than measures of women's socioeconomic status.

#### The Relevance of Gender in/Equality in Question

While the link between IPVAW and gender inequality is firm in Swedish policy (e.g., Skr, 2016/17:10), a co-existence of explanatory models for IPVAW in Sweden has repeatedly been pointed out. Thus, structural perspectives focused on gender and power dynamics exist alongside a focus on factors related to the individual, such as psychological pathology, substance abuse, or socioeconomic marginalization (e.g., Enander, 2010; Wemrell et al., 2019). Arguably, attention towards gendered power dynamics has tended to decrease or fall away in practice, with focus increasingly being directed towards risk factors or particularly vulnerable groups, and using more gender-neutral concepts and perspectives (e.g., GREVIO, 2024; Hoppstadius, 2018). A relative absence of attention to gendered power relations has thus been noted in Swedish healthcare (Pratt-Eriksson et al., 2014), judicial (Agevall, 2012; Swedish Gender Equality Agency, 2022), social service (Hoppstadius, 2018; Kjellberg, 2025), and private shelter (Lauri et al., 2023) organizations. Meanwhile, IPVAW has tended to be perceived as a structural problem in other cultures, more so than in the Swedish majority population (e.g., Karlsson et al., 2021; Wemrell et al., 2019).

Some researchers have argued for the continued importance of a feminist theoretical framework for the understanding of IPVAW, as it anchors the issue in social relationships and dynamics of power rather in attributes of individuals (DeKeseredy, 2021; Hunnicutt, 2009; Pease, 2019). Meanwhile, whereas the use of gender-neutral perspectives has the clear and crucial value of enabling attendance to intimate partner violence (IPV) against men (e.g., Nybergh et al., 2013) and in same-sex relationships (e.g., Ovesen, 2021), it can also be associated with an assumption of existing gender equality (Ekström, 2018; Wemrell et al., 2022). Such assumptions can fortify obstacles to reporting and addressing IPVAW, for example through stifling conversations about IPVAW (Clarke, 2011), creating barriers to leaving violent partners (Enander, 2010) and strengthening victim-blaming attitudes (Gottzén & Korkmaz, 2013). This indicates a need for further understanding of how gendered power dynamics can function in the context of IPVAW in Sweden.

### Aim

This study explores experiences of gender in/equality in violent relationships in Sweden, through analyzing the accounts of 23 IPVAW survivors. While this qualitative interview study does not seek to explain the Nordic paradox as such, it is pursued against the backdrop of high levels of IPVAW in a society characterized by relative gender equality. Drawing on the domains of the EIGE GEI (2017a, 2024)—work, money, knowledge, time, power, and health—we examine how these aspects of life were described in relation to the women's experiences of intimate partner violence.

## Materials and Methods

The study is based on interviews with 23 women exposed to IPVAW in Sweden. Our participants were recruited through women's shelters and municipal support units, primarily through an invitation posted in social media by a women's shelter. A few were contacted directly after referring to experiences of IPVAW in social media or in a professional context, or through snowball recruitment. They were aged 21–54 years. All had been exposed to IPVAW in heterosexual relationships, which had lasted for 1.5–23 years and were now, except in one case, over. A few women had been in more than one violent relationship. Due to our interest in the Swedish context, and to tendencies in Sweden towards understanding IPVAW as mainly occurring in other cultures (Wemrell et al., 2019), the invitation was aimed to women self-identifying as Swedish. Thirteen of the women referred to both themselves and the violent partner as Swedish. Two women had been adopted from another country, and five had some other form of non-Swedish background, although they had lived in Sweden for all or most of their lives. Of the violent partners, six had non-Nordic backgrounds. One woman had been exposed to violence by Swedish and foreign-born men.

The women had been subjected to a spectrum of degrees and types of violence. All noted instances of physical and psychological violence, and many of sexual or economic violence, or violence using information technology. Nine had been threatened with murder, and two described murder attempts. Some spoke of violence enacted through children or via support institutions. All described experiences which can be categorized as intimate terrorism (Johnson, 2008), i.e., the partner used violence to maintain control, while they did not. In most cases, the violence had developed gradually, for some not until after several years. The violence had continued after the separation for some, and in a few cases, it was still ongoing.

The interviews were conducted at Lund or Linnaeus University, over the phone or at the participant's home. They were held in Swedish and lasted for 1–2.5 hr. One follow-up interview was held. A semi-structured interview guide was used. After introductory questions about the women's life situation, they were asked to speak about the violent relationship and about their experiences of gender in/equality within it. Questions about the latter drew on the domains of the EIGE (2017a) GEI. The EIGE GEI measures of national gender gaps are not directly translatable to the individual level. In the domain of work, for example, the GEI assesses gender gaps in participation, segregation, and quality of employment. In the interviews, open questions were posed about the women's work situation, and in the analysis anything pertaining to paid work was included in this domain.

The recorded interviews were transcribed verbatim and analyzed thematically (Braun & Clarke, 2006, 2019). First, both authors read the transcripts closely and discussed initial codes. LH then conducted an inductive coding of the interviews, creating codes based on the manifest meaning of the interview excerpts, while simultaneously categorizing the material into the domains considered in the EIGE GEI. The codes were then organized into potential themes. In the next step of the analysis, MW reviewed and developed the themes and subthemes, which were then discussed and refined by both authors, in an iterative collaborative process (cf. Braun & Clarke, 2019). The thematic analysis combined elements of a reflexive and a codebook approach (Braun & Clarke, 2023), as we brought pre-established categories, i.e., the domains of the EIGE GEI, to the analysis, but not to the generation of themes and subthemes during which patterns of shared meaning were sought. In line with a reflexive thematic approach, we regard the analysis as an actively, and inevitably partially subjective, interpretative process, and in the interest of trustworthiness, we therefore engaged in close collaborative discussions about the themes and subthemes, and their grounding in our data.

Ethical approval for the study was granted by the Swedish Ethical Review Authority (Dnr 2019-00221). In line with recommendations for ethically sound research on IPV (WHO & PATH, 2005), care was taken during communication before and after the interviews to minimize any risk of participation contributing to violence recidivism. To avoid retraumatization, the interviews aimed to cohere with Hydén’s (2014) “teller-focused” approach, through using open questions and listening closely to the participants while striving to establish a relational space where they felt safe and in control. Ample space was given for the women's own stories, with follow-up questions sometimes inviting elaboration on aspects of the accounts. A manuscript draft was sent to participants who had expressed an interest in taking part in writings before publication, for respondent validation enabling any discussion about the findings (e.g., Frambach et al., 2013).

## Results

The themes and subthemes generated in the analysis (Table 1) are expressive of the women's accounts of experiences of gender in/equality and violence pertaining to the respective domains of the EIGE GEI. The themes created in relation to the domains of work, money, and knowledge are *relative socioeconomic equality*, *resources provoking violence*, and *economic violence*. The themes pertaining to the domain of time are *private sphere inequality* and *involving violence*, while those related to power are *overall subordination* and *violent enforcement*. The smaller theme generated with regard to health is *multidimensional impacts*.

**Table 1.** Thematic Analysis: Themes and Subthemes.

**Domains**	**Themes**	**Sub-themes**
Work	Relative Socioeconomic Equality Resources Provoking Violence	
Money	Economic Violence	Obstruction and Disturbance
		The Partner Controlling Income
Knowledge		Carrying the Economic Weight
		Expenses and Debts
Time	Private Sphere Inequality	Unequal Division of Domestic Labor Unequal Rules for Social Activities Gender Norms
	Involving Violence	Entailing Violence Provoking or Averting Violence
Power	Overall Subordination	The Partner as Decision-Maker
		Making Decisions and Carrying the Load
	Violent Enforcement	
Health	Multidimensional Impacts	Violence and Health Effects on Other Domains

### Work, Money, and Knowledge

#### Relative Socioeconomic Equality

While the women's status regarding education, employment and income varied in absolute terms and relative to that of their violent partners, most of them had a similar or higher status than the men. A majority of the women had a higher education than their partners, and almost half reported having earned more than them.

#### Resources Provoking Violence

Some women mentioned that their employment, income, or education triggered violence. Regarding work, some described being physically prevented from leaving home for work, or violence escalating when they advanced in their career. Concerning income, Sophie recounted her partner being provoked by her earnings:

It made him crazy that I pulled in more money than him.

Others noted that the more they pursued an education the angrier their partner became, or they described using strategies to downplay their education to evade violence. In Sanna's words:

It bothered him terribly that I had an education and a career. He didn’t think I should talk about it […] Then he was enraged.

#### Economic Violence

*Obstruction and disturbance.* Most women described forms of economic violence, many using that concept explicitly and others describing acts falling under this category. Some spoke about their partners obstructing their work or education, through forbidding them to work or study, or causing them to lose their job. Some had been prohibited from attaining a tertiary education. Jasmin recounts:

He absolutely did not want me to study. Because then I would have had a better education than him and he didn’t want that […] When I said I wanted to study, he became angry.

A few recounted losing their jobs after the partner had hindered them from going, made incessant phone calls there, or demanded that they leave early. One woman had changed employment repeatedly due to her partner acting aggressively towards her colleagues. Others had to quit their jobs when leaving the relationship or were unable to work or study for a long time due to health consequences of violence. Linn did not start her education until after the relationship, during which her study became a point of focus for psychological violence:

He said: “You are not smart enough. You know that university study is for smart people, not for the likes of you” […] He broke me down… [saying] that I’m not capable.

Although not having lost their jobs, or being literally stopped from working or studying, others described being disturbed. Some were distracted by repeated messaging while they were working. Others had to limit their work-related activities, for example, abstaining from having lunch with colleagues as any social interaction could trigger violence. Some avoided or had to cancel work travel, or described such travel as being riddled with worry, because the partner did not fulfill his childcare commitments or, as in Ingrid's case, demanded her return:

If I was away at a conference he often started calling. He just: “You have to come home now.” That's what he did. That's how he controlled me when I was travelling.

The pattern of not being able to plan work travel, as the partner could suddenly drop his child-care responsibility, was noted by one woman to having been maintained after the separation.

The women's work could also be disrupted by violence directed against equipment. One woman related, for example, how her ex-partner had broken her work computer and torn apart all the clothes she had packed for a work trip. In addition, women spent time on sick leave, due to violence, which meant periods away from work or study and loss of income.

*The partner controlling income.* Several women spoke about the partner having controlled their economic means. The degree of control varied, and could entail claiming the woman's salary, controlling her purchases or spending money from mutual accounts.

Some described having access very little money, in many cases despite working, and being required to ask the violent partner for things they needed. One woman noted that she wasn’t allowed to keep her salary in her account. Another, Julia, recounted:

I earned really well, but at the same time it felt like I never had money […] there was never anything for me and the children.

Others had access to money but could not spend it freely. Annika, for example, had a high salary but her partner decided, down to the small detail, how it was going to be used. Mia described her access to money as being clearly tied to her yielding to her partner's will:

If I didn’t do what he said or if we didn’t have sex or if I responded back at him, then I didn’t get any money and I couldn’t give the children food […] He was also very particular about not letting me have the petrol card […], he always had to get that back.

Similarly, Yvonne's partner “pushed her down economically” for example by not letting her own a car and demanding that she ask for permission when she wanted to borrow his.

*Carrying the economic weight.* Other women recounted having carried a larger, or the entire, economic weight of the mutual household. Joan's violent partner, for example, didn’t pay a crown for anything. That is, I paid for all the food and everything.

Another similarly described having paid all the household costs, including those of the partner's children, adding that this was an attempt to adapt and avoid his anger. Others refer to their partners recurringly having asked them for money. In Rebecca's words:

All the time this “I have no money” […] He has used me economically. Yes, in sum I’ve surely spent a couple of hundred thousand on him.

*Expenses and debts.* Many of the women's economic situation was affected by expenses and debts, one aspect of which was costs due to violence directed at material possessions. Maria narrates:

He tore apart my clothes and jewelry […] I had a mobile phone that he just squashed […]. Once […] *all* my outdoor garments and shoes lay cut apart […] into small pieces.

Karin's partner cut the tires of her car and stalked her to the point where she, after staying at a women's shelter, felt forced to buy a new apartment. This obviously came at a cost.

Some were placed in debt by the violent partner. Jasmin, for example, spoke about how her partner acquired economic problems through borrowing and gambling, which she had to resolve alongside managing the costs of daily life, and how she therefore took loans.

Large costs associated with the separation from the violent partner were described by some. Annika escaped, together with their child, bringing nothing but the clothes they were wearing and being left economically empty-handed:

I didn’t gain any more access to the house at all […] Still today he puts things up [for sale] in social media, my things: household items, things I’ve inherited, stuff like that.

She added that she lost a large sum of money when the house was sold. Another woman spoke of her ex-partner insisting on gaining economically at their separation, and of paying him large sums to get sole lease of their apartment. In Sofie's case, the ex-partner obstructed and prolonged the partitioning of their house, which raised her legal costs significantly.

Others noted that their ex-partners had emptied mutual bank accounts, during or after the relationship. Some referred to their ex-partners not paying alimony for, or any expenses of, mutual children, and to accepting that to avoid violence. Some were still in debt. Two had been economic exploited quite recently, for example through the forging of their signature.

In sum, while many women had a similar or higher socioeconomic status than their partners, the IPVAW affected and involved the domains of work, money, and knowledge through economic violence and, in alignment with the backlash hypothesis (cf. van Vugt & Pop, 2022; Wemrell, 2023) or with resource theory (cf. Atkinson et al., 2005; Moe & Bell, 2004), through violence being triggered by the women's resources.

### Time

#### Private Sphere Inequality

In the domain of time, the EIGE GEI measures gender differences in time spent on care activities (domestic work; care for children or others) and social activities (sporting or leisure activities; voluntary or charitable activities). Compared to the domains of work, money, and knowledge, patterns of gender inequality were more evident in this private sphere, in the women's accounts. Gender inequality was also a word used by several of the women in this context, in contrast with the previous domains about which they generally spoke more in terms of control or violence.

*Unequal division of domestic labor.* Regarding the division of domestic work, a few women stated that their ex-partners were in favor of equality, but only one related actually having shared the housework equally. All others recounted having done a larger share of this work. Jasmin estimated that she did three times as much work as her ex-partner, while Joan estimated that she did 98% of it. Some, like Linn, described having done it all:

I did everything. I was to cook for him. Clean. He has never helped me.

Regarding child-care, many participants had children with a violent ex-partner or from previous relationships. All of them recounted having taken more or all responsibility for child-care. One woman commented that her partner had never changed a diaper, while another noted that he had not spent any time alone with the child.

*Unequal rules for social activities.* Most of the women spoke about their ability to partake in social activities as being curtailed during the violent relationship. A few of them described being completely isolated, while others referred to their social lives becoming limited. Many noted that the rules applying to them were different to those of the partner, who enjoyed more freedom. In Karin's words,

He was allowed to do more than me.

Louise, who could not meet a friend for coffee, related how her ex-partner went on vacations for weeks on his own. She saw this as unfair but commented that she just had to swallow it.

*Gender norms.* Many accounts pertaining to domestic work and social activities coalesce in the partner's view of the woman's place as being in the private sphere, i.e., of him perceiving domestic labor as women's work and pushing for her to stay in the home. Mia quotes her ex-partner:

“a woman exists to be at home and take care of the home—when the hell are you going to learn that?” He thought women should know their place […]. I should be at home and serve him, quite simply, and take care of the house.

Thus, staying in and caring for the home was tied to notions of appropriate femininity.

#### Involving Violence

*Entailing violence.* The domestic work placed on the women's shoulders was not only described as unequally distributed, but often as directly tied to control and violence. Some women refer to having to be at their partners’ beck and call: to being like an “errand boy” or a “slave.” Amanda said:

It really felt like I was meant to just be like a slave for him and do what he wanted. Cook, clean, take care of the home […] It was like living in a prison.

Being required to do all the domestic work was thereby described by some as a form of emotional repression or violence in itself, or, as expressed by Ylva, a form of punishment:

I think it was really about him wanting to punish me in some way […] That I was going to carry that work burden. Yes, so that it would be difficult for me.

While the women were typically assigned the large share of child-care, several referred to parental responsibility being used as a “means of power” or control. For example, partners changing plans or relinquishing child-care tasks could, as noted, limit and obstruct the women's work. Moreover, some women's child-care responsibility was drawn into conflict as ex-partners who had previously not involved themselves in childcare fought for sole custody.

Regarding social activities, the women's accounts of being pressured to limit their actions and relationships, including with friends and family, mirror a well-known feature of IPVAW (e.g., Penttinen, 2024). Thus, stories of partners resisting, obstructing, or forbidding women's social activities refer to aspects of their violence.

*Provoking or averting violence.* Several women spoke about violence being triggered by them not doing sufficient domestic work, as perceived by their partners, or by their resistance to their partner's demands. Some, like Sara, referred to psychological violence focused on this realm and on the women's value:

If you don’t do it (…) then you just get beaten more or hear more that you’re worthless.

Consequentially, doing domestic work was described by some as a way of avoiding violence. Yvonne, for example, worked to keep her partner in a good mood:

I could make every utmost effort […] You know, you did everything for there to be peace and quiet […] That was probably more about survival.

Linn spoke about having made up domestic chores, as her partner left her alone when she was occupied with the household. Regarding child-care, women referred to having done everything to avoid the partner being disturbed, as that could trigger anger or violence.

Concerning social activities, the link between women's social interactions, or initiatives towards such, and violent responses from their partners was very clear in several accounts. One woman related having received her first slap in the face when she said she missed her parents and wanted to visit them, while another noted that the more she tried to achieve gender equality by claiming freedom of movement, the angrier her partner became. Ylva said:

He wanted me to just be at home and fix things there, cook and stay there […] the more I tried to break loose and become my own, then he became … everything got worse.

Many spoke about avoiding social engagements altogether, or keeping them secret, to avoid violence. Accounts included, for example, fear of mentioning having met a friend for coffee, anxiety about how to handle party invitations, or the distress of giving away a pet because the partner disapproved of the time spent with it, alongside the fear that he might end its life. Some added that they wanted to avoid situations where the ex-partner could become aggressive in public, or where they might have to conceal or explain the violence to others.

In sum, while the women generally described an unequal division of housework and social activities, their accounts highlight how these inequalities could involve violence, and how resisting them could provoke it.

### Power

#### Overall Subordination

In the domain of power, the EIGE GEI measures gender gaps in the share of representatives in key political, economic, and social organizations. In the present private sphere context, we look at how the women spoke about decision-making and power in the violent relationship. All referred to their partners as claiming or being in a position of dominant power.

*The partner as decision-maker.* According to most of the women, their partners held the main decision-making power in the relationship. Nine said the partners had more decision-making power, while 10 stated that he made all the decisions. In Carolina's words:

It was on his terms, that's how it had to be.

In some accounts, the violent partner claimed that power explicitly. Linn, for example, recounted her ex-partner's response when she tried to negotiate everyday decisions:

You are married and you shall obey me.

In others, women yielded to the men's will. Some spoke about a surface appearance of mutual decision-making, although the violent partner's will was decisive in practice. Rebecca says:

There was an imagined belief that we were equals and that we had an equal amount of power. But in his conversations or in his way of being he always managed to knock me or what I was thinking over […] So that things would turn out the way he wanted.

Rebecca, like others, did not fully realize the extent to which she adapted to her partner's will, until after the relationship is ended.

The partner's decision-making was in some cases very far-reaching. Mia could not wear, read, or eat what she wished. Annika was not allowed to eat when or what she wanted, and sometimes not at all. Thus, their partners micro-managed their lives down to the detail.

*Making decisions and carrying the load.* A few recounted having made plenty of decisions in the violent relationship, connecting this to the partner abstaining from taking responsibility. Yvonne noted that she was responsible for fixing and deciding on practical matters, whereas her partner “escaped it.” Jasmin similarly related having planned and executed things, while her partner did not contribute:

I was the one who managed things, absolutely […] Yes, it was like living with a big child [laughs]… that is, I was the one who had to manage daily life.

Here, decision-making was described as carrying the responsibility for daily life. This did not equate to having power, as the women still expressed a general sense of subordination.

#### Violent Enforcement

While all the women spoke of their partners as being in a position of dominant power in the relationship, many referred to violence used as a means of enforcing or upholding that position. Some recounted that their acts of resistance towards the partner—drawing limits, putting their foot down, or speaking back—provoked violence. Violence was also described as being used to enforce the partners’ decision or will. Marie, for example, speaks about how when she expressed a differing opinion, her partner claimed power by becoming violent:

When his arguments had run out, he resorted to this and then I became quiet and the discussion was over […] That's how he took the power. He took back the power when he became violent.

Correspondingly, many noted that the reason why they yielded to the partner's will was to avoid the threat of violence. In Karin's words:

[I] chose to follow his line. To escape [violence].

Decision-making could also integrate psychological violence. For example, Annika described how her partner asked for her opinions or suggestions, only to “carefully shoot them down.”

Some women described the drives towards violence and power as being closely linked. Thus, Amanda spoke about violence as being driven by a need or desire for power:

It's some kind of need for power. That is, that feeling of power. That's what it's about.

Jasmine stated that the presence of violence renders equality impossible. If there is violence, she said, there is no equality. Similarly, Ellinor placed control in opposition to equality, noting that her ex-partner wanted the former and not the latter:

He didn’t want a relationship of equality […] he wanted to have control over me […] If I became too independent or strong in some way, then he had a need to push me down.

In sum, while the division of decision-making varied, all the women described their partners asserting or holding a position of dominant power in the relationship. This power was typically described as being maintained through violence, including the threat of it.

### Health

#### Multidimensional Impacts

The EIGE GEI measures gender gaps in health outcomes, behaviors, and care access. In the interviews, such differences were not a main focus. The smaller theme pertaining to this domain includes health effects of IPVAW and such effects spilling over into other domains.

*Violence and health.* Women described both somatic and mental health effects of the violence they were subjected to, including depression, anxiety, posttraumatic stress, incontinence, and suicidal tendencies. Accounts of violence having a direct impact on health include partners neglecting them after they had broken a leg or given birth, or severely limiting their food intake. Many health issues remained after the separation. Moreover, while some spoke positively about the support they had received from healthcare and social services, others described such help as limited and in some cases as exacerbating their mental ill-health. The latter included traumatization being interpreted in terms of lacking parental ability potentially motivating loss of custody.

*Effects on other domains.* Health consequences were described as having affected other areas of life. Regarding the domain of knowledge, Carolina spoke about the violence impacting her studies:

My anxiety was so tangible. Those times that I went to lectures I had to leave the lecture hall and go to the bathroom to throw up. I was feeling so bad.

After the violence had caused a long break in her education, Malin returned to university when relationship had ended, but she still had to handle posttraumatic stress while studying.

Most of the women describe having spent periods on sick leave, which can obviously have implications in the domains of work, money, and knowledge. Ellinor, who was on sick leave for 6 months, noted that her economy, and not that of the partner, took a blow. She adds that mental health effects of the violence affected her social life, i.e., the domain of time:

I’ve always been quite social. But that was smashed apart […] I was broken down somehow […] I lost a lot of friends […] and had to build everything from scratch.

In sum, the women spoke of many health effects of the IPVAW, that impacted other domains.

## Discussion

This interview study examined women's experiences of gender in/equality in violent intimate relationships in Sweden. Drawing on the six domains considered in the EIGE GEI (2017, 2024), we investigated how the women described experiences related to work, money, knowledge, time, power, and health, in their violent relationships. In their accounts, all these domains were influenced by and involved in IPVAW, creating pressure towards subordination in ways that may not be congruent with or readily apparent in measures of gender equality. Thus, while unable to provide an explanation for the Nordic paradox, or to compare aspects of gender in/equality in violent heterosexual relationships to those in non-violent ones, this study indicates that gendered power dynamics remain central to IPVAW in Sweden, including among relatively socioeconomically empowered women.

### Work, Money, and Knowledge: Relative Equality and Violence

Although the women's socioeconomic status varied, the accounts indicated relative gender equality overall, with several women having attained higher positions than their partners with regard to income, education, or employment. Still, these socioeconomic factors were linked to IPVAW, through economic violence and violence triggered by the women's resources.

While previously often overlooked, economic IPVAW has in recent years been given increasing attention, including in Sweden (Bruno et al., 2025; Eriksson & Ulmestig, 2021; Fernqvist et al., 2024) and other Nordic countries (Kaittila et al., 2024). Economic violence is often divided into three categories (e.g., Postmus et al., 2012), all of which be seen in this study. Economic exploitation occurs when the offender uses the victim's resources or causes her to be placed in debt, economic control entails limiting the victim's use of her resources, and employment sabotage consists of hindering her work (cf. Kaittila et al., 2024). Emphasizing that economic violence often continues after separation (cf. e.g., Fernqvist et al., 2024), Kaittila et al. (2024) note that destroying possessions, creating costs through prolonging divorce, refusing to pay bills, withholding child support, and denying access to property are various types of economic violence, also found in the accounts of our participants.

This highlights not only that economic violence can take different forms, but that such violence can also be directed against relatively socioeconomically privileged women. This is worth noting against the backdrop of the Nordic paradox, and as women in more privileged positions or who self-identify as strong have shown particular resistance to identifying as IPVAW victims and have been less likely to be perceived, and receive social or legal support, as such (Agevall, 2012; Jarnkvist & Brännström, 2019). This is while women with higher income have rarely been in focus in efforts to address IPVAW, in Sweden (Ericsson, 2020) or internationally (e.g., Wang & Sekiyama, 2024).

Women's accounts of their income, education, or employment provoking violence are in line with resource theory (cf. Atkinson et al., 2005; Moe & Bell, 2004) and with the backlash hypothesis (cf. Bjelland, 2014; Ericsson, 2020; van Vugt & Pop, 2022). It is likely that ameliorative effects were also present, as access to resources lower barriers to leaving violent relationship (e.g., Gómez-Casillas et al., 2023), which most of our participants had done. Still, it should be emphasized that such resources can be severely curtailed by economic violence (also Moe & Bell, 2004). While the development towards socioeconomic gender equality can likely involve an interplay of ameliorative and backlash effects (cf. Gómez-Casillas et al., 2023; Kosakowska et al., 2020), and this study cannot say anything about the relative importance of such effects on IPVAW at the population level, our results do indicate the existence of backlash dynamics and women's resources provoking violence. Moreover, as noted by Moe and Bell (2004), whether or not IPVAW is specifically targeted at women's employment, it can severely impede their ability to work, including among women with well-paid careers.

### Time and Power: Inequality and Violence

Patterns of gender inequality were clearer, and named as such by participants, in accounts tied to the more private domains of time and power, which also involved violence.

With regards to the domain of time, and to domestic work, all the women except one related having done most, or all, this labor within the violent relationship. This is not unique to violent relationships, as women in Sweden often bear a greater share of domestic work (e.g., Nyman et al., 2018; Statistics Sweden, 2022). However, as also seen in this study, links between IPVAW and traditional views on women as tied to the domestic sphere have been observed. In a study from Finland (Penttinen, 2024), for example, victims noted that their partners had wanted to train them to be stereotypical subordinate wives, attending to the partner's very wish and always being available. This resonates with our participant accounts, which speak not only of an unequal division of domestic labor tied to traditional gender norms, but of breaking with such demands or norms triggering violence.

Inequality is also found in accounts of limited social activities. Restricted freedom of movement is a well-documented feature of IPVAW (e.g., Penttinen, 2024), and resistance to such restrictions was among factors noted by the participants to provoke violence (cf. Wemrell, 2023). Moreover, many accounts pertaining to social activities as well as domestic work coalesce in the image of the woman's place being in private sphere. That is, many women referred to violent partners forcefully pressing them to conform to that role or norm.

Concerning the power domain, all the women noted that their violent partner claimed a position of dominant power in the relationship. This was often reflected in the partner being the main decision-maker, although in some cases women made decisions as part of carrying the responsibility to make daily life work. Either way, violence was typically described as a way of claiming, enforcing, or maintaining the partner's position of power.

This drive towards claiming power can be related to Gómez-Casilla et al.'s (2023) finding that while ameliorative effects can be shown in relation to some domains of the EIGE GEI, such as money or work, backlash effects occurred when men's status was lower overall. Relatedly, IPVAW perpetrators in Sweden have described their violence as triggered by women being bossy or claiming power in the relationship (Edin & Nilsson, 2014). This is in line with international studies where men referred to women questioning their authority or being insubmissive as reasons for being violent against them (De Coster & Heimer, 2021). It has also been noted that changing gender norms can contribute to IPVAW, as ideals of equality co-exist with traditional ones (Gottzén & Korkmaz, 2013; Wemrell et al., 2022).

Penttinen (2024) points to the instilling of a sense of powerless in victims as a central aspect of IPVAW. Some of her examples of how this is done, such as controlling food intake, threatening to kill pets and withholding child-care, correspond with our participants’ accounts. These also resonate with Stark’s (2023) description of coercive control, as the micro-regulation of women's behavior pressuring them to conform to traditional gendered expectations. Stark argues that coercive control is likely more common in more gender-equal countries, as it can serve to contain the effects of women's increased independence. Thus, he argues, the very socioeconomic opportunities that enable women's free mobility provide a heightened incentive for coercive control sustaining their containment in the private sphere. This paradox (Stark, 2023) is reminiscent of the Nordic one (cf. Pease, 2019).

Stark’s (2023) description of coercive control as maintaining a gap between women's opportunities or rights and their ability to enjoy them can perhaps be related to women's equal political power not being guaranteed by parity in positions of power, as women in politics are exposed to more violence than men in Sweden (Håkansson, 2019). This is also in line with the backlash hypothesis, and the argument that structural changes in the public sphere do not necessarily align with ideological or cultural ones (Hunnicutt, 2009; Pease, 2019).

Returning to the private sphere (cf. Ertürk, 2007), our results underline that gender inequalities in the domestic arena, closely tied to IPVAW, can be present including in relationships where the woman occupies a relatively privileged socioeconomic position.

### Health: Multidimensional Impacts of Violence

In the health domain, women's accounts pointed to various long- and short-term health effects of violence, not rarely continuing after the relationship ended and on occasion through contacts with support organizations. Some accounts referred to health effects of violence spilling over into the domains of work, knowledge, income, and time. Although not mentioned in these accounts, the domain of power can likely also be affected by ill-health.

Correspondingly, research has indicated the interconnectedness of effects of IPVAW in different areas of life, including after separation (e.g., Fernqvist et al., 2024). Economic violence (money) can be tied to effects on children, child-care (time), and employment (work) (Kaittila et al., 2024), while health impacts of violence can affect income (money), employment (work), and education (knowledge), through sick-leave as well as physical or cognitive impairment (e.g., Klencakova et al., 2023: Penttinen, 2024). Social isolation and economic difficulties caused by IPVAW can also increase the risk of future ill-health.

Hinting at the potentially totalizing nature of IPVAW, which Penttinen (2024) compares to being held in captivity and which relates to Stark’s (2023) notion of coercive control, this points to the interweaving of effects of IPVAW in domains of the EIGE GEI. Meanwhile, some women's accounts of revictimization experienced in encounters with societal IPVAW support structures (cf. e.g., Pratt-Eriksson et al., 2014) align with the understanding of IPVAW as a structural more so than, or as well as, an individual-level issue.

### Complex Expressions of Power: Implications

In line with EIGE's (2017b) observation that IPVAW can cause or maintain gender inequality despite gender equality policy in other spheres, and with the clear linkage of IPVAW and gender inequality found in Sweden's gender equality goals (Skr, 2016/17:10), this study indicates that gendered power dynamics remain highly relevant to IPVAW in Sweden, although other causative factors can surely contribute. We thereby side with research (e.g., Hunnicutt, 2009; Pease, 2019) and policy (Council of Europe, 2011) affirming the continued centrality of focusing on gendered, societal power relations for the understanding and addressing of IPVAW, including in Sweden (cf. GREVIO, 2024).

The study shows that gendered power dynamics can take many forms, in a range of life domains, within violent relationships. Importantly, such expressions of power can look quite different, although the underlying relationship of dominance is basically the same. In the domain of money, for example, the partner could take control over the woman's resources, or let her carry the economic weight of the household. In the domain of power, the partner could claim all decision-making, or leave the responsibility for maintaining daily life, including for making decisions, to the woman. Relatedly, Montminy (2005) notes that psychological IPVAW can take active forms, i.e., things being said or done, as well as passive ones, i.e., things *not* being done, including neglecting or avoiding responsibility. Understanding such complexities can be crucial for measuring, inquiring about, and addressing IPVAW.

In line with suggestions by IPVAW professionals (Wemrell et al., 2022) that measures of gender equality such as the EIGE's GEI likely do not reflect all relevant aspects of in/equality, our results point to power dynamics which would not necessarily be visible in population-level assessments of gender equality. While the accounts point to varying but relative socioeconomic gender equality overall, they also show women's resources provoking IPVAW, including economic violence, and private sphere inequalities linked to violence. While a few women spoke of relationships having been largely gender equal in some domains, all converged in their descriptions of the violent partner claiming the main power. While it is commendable that the EIGE GEI includes private sphere measures in the domain of time (cf. Schmid & Elliot, 2023), although the name of the domain still renders aspects such as domestic labor relatively invisible, this underscores that the GEI does not express all aspects of in/equality of relevance for IPVAW.

Against this background, assuming or idealizing gender equality in Sweden, in relation to IPVAW, appears problematic. This is not least the case as gender equality in Sweden has recently decreased (EIGE, 2024), arguably related to processes of dismantling the welfare state, in the context of which the favoring of Sweden as a gender equality policy model has been critiqued (Schmid & Elliot, 2023). Meanwhile, signs of a decreasing support for (Kosakowska et al., 2020; Swedish Gender Equality Agency, 2021) and backlash against gender equality can be seen in Sweden (Wemrell et al., 2022) as elsewhere (EU, 2018).

Moreover, and as noted, recent decades have seen increasing attention to IPVAW in particularly vulnerable groups and in relation to various risk factors, as well as gender-neutral terms and approaches. While it is very important to study and address different forms of IPV against different victim groups, not least with a focus on the relevance and nature of power dynamics (e.g., De Coster & Heimer, 2021; Pease, 2019), we argue for the importance of not losing sight of gendered power relations when addressing IPVAW. This is while, as also noted, a relative absence of focus on gendered power relations has been noted in Swedish support institutions (e.g., Hoppstadius, 2018; Kjellberg, 2025; Swedish Gender Equality Agency, 2022) including the increasingly present privately run women's shelters (Lauri et al., 2023). Worth noting here is that recent reforms in Swedish family policy, associated with assumptions of and aiming to promote gender-equal parenting, may contribute to the facilitation of IPVAW including financial control (Bruno et al., 2025; Fernqvist et al., 2024). In the handling of child custody cases, IPVAW has been made invisible or understood in terms of mutual conflicts or difficulties in parental cooperation. This can have effects including the enabling of post-separation violence and requiring children to be cared for by violent fathers (Fernqvist et al., 2024; Kjellberg, 2025).

Challenges to identifying, reporting, and addressing IPVAW due to assumptions of gender equality can include hindering conversations about IPVAW (Clarke, 2011), triggering feelings of shame, stupidity, or self-blame among victims, and making self-identification as such a victim more difficult (Penttinen, 2024), strengthening victim-blaming attitudes (Gottzén & Korkmaz, 2013), and creating barriers to leaving violent partners (Enander, 2010; Gottzén, 2013). In addition, the woman's sense of being the stronger person in the relationship has been noted to not only make it harder to identify and handle IPVAW, but also to contribute to the triggering and unfolding of the violence (Wemrell, 2023; cf. Donovan & Hester, 2015). The violent partner's adherence to gender equality ideals or a feminist identity can also make it difficult to see and understand IPVAW (Wemrell, 2023).

This, again, highlights the importance of addressing gendered power dynamics, not least in cultural or ideological realms (Hunnicutt, 2009; Walby, 1990). The importance of addressing gender norms, and of attitudes legitimizing violence, has been emphasized in Sweden (e.g., Agevall, 2012; Skr, 2016/17:10) and internationally (e.g., WHO, 2007). Arguments have been made for normalizing partnerships where women have a higher income or education (van Vugt & Pop, 2022) and supporting men's engagement in caring roles (Pease, 2019). Moreover, some IPVAW survivors pointed to their partner's perceived feeling of subordination as contributing to violence (Wemrell, 2023), which underlines the importance of counteracting men feeling threatened by gender equality. Such efforts may include increasing knowledge about men's mental health, supporting men's capacity to be vulnerable and masculinity norms inconducive to violence (Heise et al., 2019; Krumm et al., 2017; Pease, 2019), as well as mitigating understandings of gender equality in terms of a zero-sum game where one group's gain is the other's loss (cf. Kosakowska et al., 2020), in favor of emphasizing the benefits of gender equality and non-violence for all (Flood, 2019).

Such efforts should be accompanied by an overarching focus on power structures (Pease, 2019), enabling and including awareness that ameliorative and backlash processes may intermingle (e.g., Whaley & Messner, 2002) and that the risk of backlash dynamics or coercive control can be substantive in the presence of women's relative socioeconomic empowerment or where dominant masculinity is perceived to be threatened (De Coster & Heimer, 2021). This is of relevance in Sweden, and, because of the importance of understanding the Nordic paradox due to potential implications for IPVAW policy and prevention (Hardesty & Ogolsky, 2020), elsewhere.

### Limitations

While investigating experiences of gender in/equality in violent relationships in Sweden, this qualitative interview study cannot provide any explanation for the Nordic paradox. Nor can it say anything about power dynamics in IPVAW as compared to those in IPV against men or in same-sex relationship, or compared to non-violent relationships.

The concept of the Nordic paradox, and the research focus on gender in/equality, was outlined in the invitation to potential participants, and it is possible that women with a proclivity towards thinking about their experiences of IPVAW in terms of gender in/equality were therefore more likely to take part in the study. It is also possible that the participants were more socioeconomically privileged than IPVAW survivors in Sweden overall, which may have increased the likelihood of experiences of backlash dynamics or resources provoking violence. These issues do not, however, detract from the relevance of our analysis.

The trustworthiness of our results was ensured through the authors’ collaborative analysis, encompassing meticulous discussion of the themes and their grounding in the data, and by respondent validation (Frambach et al., 2013).

## Conclusion

This study of women exposed to IPVAW in Sweden, drawing on the domains considered in the EIGE GEI, found that the violence was described as having influenced and involved all these domains of the women's lives. Relative socioeconomic gender equality co-existed with women's resources provoking IPVAW, including economic violence, and private sphere inequality involving violence. We conclude that despite Sweden being one of the most gender-equal nations in the world, gendered power dynamics here remain central to IPVAW. This should be an essential concern in efforts to develop effective strategies for addressing and preventing such violence.
